# Shagreen Patch in Tuberous Sclerosis Complex at High-Frequency Ultrasound

**DOI:** 10.3390/diagnostics15243168

**Published:** 2025-12-12

**Authors:** Corrado Tagliati, Ximena Wortsman

**Affiliations:** 1 AST Ancona, Ospedale di Comunità Maria Montessori di Chiaravalle, Via Fratelli Rosselli 176, 60033 Chiaravalle, Italy; 2 Department of Dermatology, Faculty of Medicine, Universidad de Chile, Av. Independencia 1027, Independencia, Región Metropolitana de Santiago, Santiago 8380453, Chile; xworts@yahoo.com; 3 Department of Dermatology, School of Medicine, Pontificia Universidad Catolica de Chile, Av. Libertador Bernardo O’Higgins 340, Región Metropolitana de Santiago, Santiago 8331150, Chile; 4 Institute for Diagnostic, Imaging and Research of the Skin and Soft Tissues (IDIEP), Lo Fontecilla 201 of 734 Las Condes, Región Metropolitana de Santiago, Santiago 7591018, Chile; 5 Department of Dermatology and Cutaneous Surgery, Miller School of Medicine, University of Miami, 1120 NW14th St Ste 9, Miami, FL 33146, USA

**Keywords:** shagreen patch, tuberous sclerosis complex, high-frequency ultrasound, dermis, skin, hypoechoic, thickening

## Abstract

We present a case of a 19-year-old female patient with tuberous sclerosis complex and a shagreen patch on her left dorsal region. Decreased echogenicity and increased thickness of the dermis were shown by high-frequency ultrasound. To the best of our knowledge, this is the first reported ultrasound image of a shagreen patch.

**Figure 1:**
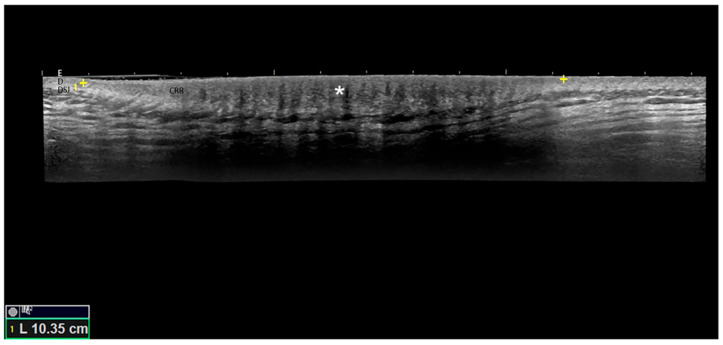
Greyscale, longitudinal panoramic view ultrasound image of a shagreen patch in tuberous sclerosis complex in the left dorsal region. Ultrasound examination was performed using a Logic E9 XD Clear ultrasound device (Logiq E9XD, General Electric Health System Clear, Waukesha, WI, USA), using a linear probe with a frequency that ranges from 8 to 18 MHz. Notice the focal zone (*) with decreased echogenicity and increased thickness of the dermis (D), which represents the collagen rich-region (CRR) between the epidermis (E) and dermal–subdermal junction (DSJ). The patient had histologic confirmation after surgical excision performed due to significant distress related to the large size and aesthetic reasons. The patient had facial angiofibromas and ungual fibromas, too. A shagreen patch is one of the major diagnostic criteria for tuberous sclerosis complex, with the others being hypomelanotic macules, angiofibroma or fibrous cephalic plaque, ungual fibromas, multiple retinal hamartomas, multiple cortical tubers and/or radial migration lines, subependymal nodules or giant cell astrocytoma, cardiac rhabdomyoma, lymphangiomyomatosis, and angiomyolipomas. A combination of two major clinical features meets the criteria for a definite diagnosis, apart from the combination of lymphangiomyomatosis and angiomyolipomas without other features [1]. Tuberous sclerosis complex is a rare autosomic dominant genetic disorder with an estimated incidence ranging from 1 in 6000 to 1 in 10,000 live births, characterized by the development of hamartomas in multiple organs caused by mutations in the TSC1 (hamartin) or TSC2 (tuberin) genes [2,3,4]. A shagreen patch is a type of connective tissue nevus which previous studies reported in approximately 21% to 83% of patients with tuberous sclerosis complex, and it often appears in the first decade [5,6]. Dark-skinned people seem to have an increased probability of a shagreen patch [7]. It is usually an irregular, firm plaque with a rough texture located on the back, and lesions can be subdivided into small (1 to <4 cm), medium (4 to <8 cm), and large (≥8 cm) ones [5]. It corresponds histologically to collagenoma (or collagenic hamartoma), with relatively acellular and hyaline collagen bundles that replace the dermis, and extends down to the hypodermis [8]. Some very large shagreen patches were previously described in the literature [5,9]. A previously published case described shagreen patch dermoscopic patterns, such as reddish-brown strands that correspond to papillomatosis, as well as dense collagen bundles in the dermis associated with regularly spaced white dots that correspond to eccrine sweat duct openings on the skin surface [10]. Another article showed that shagreen patch dermoscopy can reveal white/yellow structureless areas and reticular vessels [11]. However, to the best of our knowledge, this is the first ultrasound image of a shagreen patch.

## Data Availability

Data are contained within the article.

## References

[1] Northrup H., Aronow M.E., Bebin E.M., Bissler J., Darling T.N., de Vries P.J., Frost M.D., Fuchs Z., Gosnell E.S., Gupta N. (2021). Updated International Tuberous Sclerosis Complex Diagnostic Criteria and Surveillance and Management Recommendations. Pediatr. Neurol..

[2] Jurca A.A., Hodisan R., Jurca A.D., Severin E., Jurca S., Trandafir A., Ilias T., Vesa C., Jurca C.M. (2025). Tuberous Sclerosis Complex: A Case Series from a Romanian Genetics Center and a Review of the Literature. J. Clin. Med..

[3] Steiner C.E., Puzzi M.B., Marques-de-Faria A.P., de Oliveira Sobrinho R.P., Gil-da-Silva-Lopes V.L., Moreno C.A. (2025). A Series of Patients with Genodermatoses in a Reference Service for Rare Diseases: Results from the Brazilian Rare Genomes Project. Genes.

[4] Rodríguez-Torres D.A., Arenas-Estala J., Sánchez-Cortés R.G., Dávila-Escamilla I.V., Nieto-Sanjuanero A., López-Uriarte G.A. (2025). Prenatal Diagnosis and Management of Tuberous Sclerosis Complex with Cardiac Rhabdomyoma: A Case Report Highlighting the Role of Sirolimus and Postnatal Complications. Diagnostics.

[5] Bongiorno M.A., Nathan N., Oyerinde O., Wang J.A., Lee C.R., Brown G.T., Moss J., Darling T.N. (2017). Clinical Characteristics of Connective Tissue Nevi in Tuberous Sclerosis Complex With Special Emphasis on Shagreen Patches. JAMA Dermatol..

[6] Ebrahimi-Fakhari D., Meyer S., Vogt T., Pföhler C., Müller C.S.L. (2017). Dermatological manifestations of tuberous sclerosis complex (TSC). J. Dtsch. Dermatol. Ges..

[7] Pounders A.J., Rushing G.V., Mahida S., Nonyane B.A.S., Thomas E.A., Tameez R.S., Gipson T.T. (2022). Racial differences in the dermatological manifestations of tuberous sclerosis complex and the potential effects on diagnosis and care. Ther. Adv. Rare Dis..

[8] Cascarino M., Leclerc-Mercier S. (2021). Histological Patterns of Skin Lesions in Tuberous Sclerosis Complex: A Panorama. Dermatopathology.

[9] Kuntoji V., Bhagwat P.V., Kudligi C. (2018). Giant Shagreen Patch in Tuberous Sclerosis Complex. Indian Dermatol. Online J..

[10] Gundalli S., Ankad B.S., Ashwin P.K., Kolekar R. (2015). Dermoscopy of shagreen patch: A first report. Our Dermatol. Online.

[11] Plázár D., Meznerics F.A., Pálla S., Anker P., Farkas K., Bánvölgyi A., Kiss N., Medvecz M. (2023). Dermoscopic Patterns of Genodermatoses: A Comprehensive Analysis. Biomedicines.

